# The development and validation of an instrument to measure the quality of health research reports in the lay media

**DOI:** 10.1186/s12889-017-4259-y

**Published:** 2017-04-20

**Authors:** Dena Zeraatkar, Michael Obeda, Jeffrey S. Ginsberg, Jack Hirsh

**Affiliations:** 1 Department of Health Research Methods, Evidence, and Impact, McMaster University, 1280 Main Street West, Hamilton, ON L8S 4K1 Canada; 2 Department of Family Medicine, Queen’s Univeristy, Kingston, Canada; 3 Department of Medicine, McMaster University, Hamilton, Canada

**Keywords:** Health information, Health research reporting, Health media reports, Quality assessment, Scale development, Psychometrics

## Abstract

**Background:**

The media serves as an important link between medical research, as reported in scholarly sources, and the public and has the potential to act as a powerful tool to improve public health. However, concerns about the reliability of health research reports have been raised. Tools to monitor the quality of health research reporting in the media are needed to identify areas of weakness in health research reporting and to subsequently work towards the efficient use of the lay media as a public health tool through which the public’s health behaviors can be improved.

**Methods:**

We developed the Quality Index for health-related Media Reports (QIMR) as a tool to monitor the quality of health research reports in the lay media. The tool was developed according to themes generated from interviews with health journalists and researchers. Item and domain characteristics and scale reliability were assessed. The scale was correlated with a global quality assessment score and media report word count to provide evidence towards its construct validity.

**Results:**

The items and domains of the QIMR demonstrated acceptable validity and reliability. Items from the ‘validity’ domain were negatively skewed, suggesting possible floor effect. These items were not eliminated due to acceptable content and face validity. QIMR total scores produced a strong correlation with raters’ global assessment and a moderate correlation with media report word count, providing evidence towards the construct validity of the instrument.

**Conclusions:**

The results of this investigation indicate that QIMR can adequately measure the quality of health research reports, with acceptable reliability and validity.

**Electronic supplementary material:**

The online version of this article (doi:10.1186/s12889-017-4259-y) contains supplementary material, which is available to authorized users.

## Background

The public obtains medical information from television, newspapers, and online sources. The media provides an important link between medical research, as reported in academic sources, and the lay public. Seeking health information in the media has become increasingly common, as evidenced by survey data indicating that nearly a quarter of Canadians used the internet to obtain health information in the year 2000 [1], a rate which has most likely grown over the past decade and a half. Recently, increased attention has been paid to the medical content published in the lay media, with the rise of initiatives such as Health News Review (HealthNewsReview.org), a website which critically reviews medical stories in the U.S. news, and various health website accreditation programs, such as the Health On the Net Foundation (www.hon.ch/) and URAC (www.urac.org/) which provide quality accreditation for websites following an application and screening procedure and MEDCIRLCE and MEDCERTAIN (www.medcircle.org/) which aim to develop technologies to guide consumers towards trustworthy health information [2].

Good quality reporting of health research in the lay media can help set a productive health policy agenda, increase society’s collective awareness of pressing health problems, and positively influence the public’s day-to-day health behaviors (e.g., undergoing preventative screening, pursuing a healthy diet and exercise, and promoting smoking cessation) [3–5]. Despite the valuable potential of the media as a public health tool, concerns have been raised about its reliability [1, 6–12]. For example, Haneef and colleagues (2015) identified at least one example of spin or misrepresentation of study findings in 88% of American, British, and Canadian media reports on studies of medical interventions. Given the impact health-related media reports can have, ensuring that they are of high quality is of critical importance. Misinformed readers may have heightened concerns or expectations about medical interventions which may lengthen, multiply, or complicate medical consultations and generate inappropriate health behaviors and requests for medical treatment, thereby increasing healthcare spending. Although the importance of reliable health journalism is well recognized, no empirical investigation has yet been undertaken to evaluate the quality of health research reporting in the Canadian media. Furthermore, initiatives such as Health On the Net Foundation are not aimed specifically at evaluating information about health research. This may be partly due to the paucity of available measurement tools, which has potentially stalled needed assessment and surveillance. To address this issue, we developed the Quality Index for health-related Media Reports (QIMR; Additional file 1). The objective of this paper is to describe the development and preliminary validation of the QIMR for evaluating the quality of health research reports published in the Canadian media.

## Methods

**Fig. 1:**
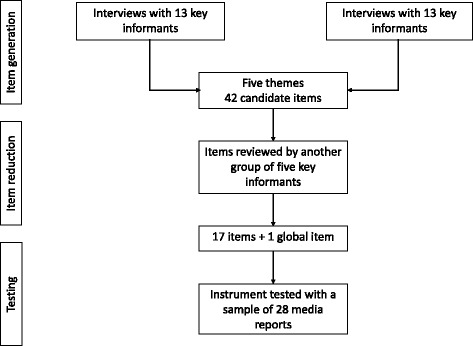
The development and testing process of the QIMR

This study was undertaken in two phases. First, the QIMR was developed through literature searches and consultation with key experts. The reliability and validity of the QIMR were subsequently tested with a sample of media reports. The development and testing process of the QIMR is presented in Fig. 1. This study was exempt from ethics review by the Hamilton Integrated Research Ethics Board.

### Literature search

A literature search was undertaken to identify existing instruments for the evaluation of the quality of health-related media reports. MEDLINE and EMBASE were searched through the Ovid interface using key terms related to health education, patient education, and journalism. No restriction was placed on publication year. The search yielded Oxman et al.’s (1993) Index of scientific quality (ISQ) for health-related news reports [13]. Given the limited yield, the search was expanded to also include instruments developed for the assessment of health information outside of the media*.* Two additional instruments were identified: Ensuring Quality Information for Patients (EQIP) and DISCERN [14, 15]. The three instruments were assessed for face and content validity.

A number of items on the ISQ were deemed to be irrelevant to the reliable communication of health research in the media. For example, when the ISQ was tested with a sample of media reports, item 1, which asks whether it is clear to whom the information presented in the media report applies, was found to be irrelevant in most cases. Discussion with key informants, such as journalists and researchers, also revealed item 5, which queries whether the media report communicated a clear and well-founded assessment of the precision of estimates, to be irrelevant. Key informants expressed that precision and confidence intervals were topics beyond the scope of what could be reasonably expected of a lay audience with no background in health research to appreciate. It was suggested that it may be sufficient for health journalists to acknowledge the possibility of false positive findings in qualitative terms as opposed to quantifying the likelihood. Finally, items of the ISQ are not organized within domains, thus limiting its capacity to identify specific areas of strength and weakness in health research reports. Unlike the ISQ, the EQIP and DISCERN were developed to evaluate the quality of health information written for patients and thus demonstrate poor content relevance to health research reporting.

To empirically evaluate the quality of health research reporting in the Canadian media, a new measure was developed that specifically addressed the content validity problems of the ISQ, EQIP, and DISCERN.

### Item generation

To guide item generation, informal, semi-structured interviews were conducted with key informants, which included Canadian health journalists and medical researchers from the departments of Medicine and Clinical Epidemiology & Biostatistics at McMaster University. All health journalists held positions at Canadian newspapers and specialized in science and health reporting. All medical researchers were either full-time medical or epidemiology professors or medical doctors pursuing further graduate education in epidemiology at the doctoral level. Key informants were asked to describe high and poor quality health research reporting. They were also asked to provide examples of high and poor quality media reports and explain their strengths and shortcomings. A total of 13 interviews were conducted with six health reporters and seven health researchers, at which point it was believed that content saturation was reached. Content generated from these interviews was organized into five themes (background, sources, results, context, validity), which were then used to guide item generation.

Forty-two candidate items were generated by a working group of three researchers (DZ, JSG, JH) who were not part of the group of key informants consulted in the previous step. Although the ISQ, EQIP, and DISCERN were found to have poor content relevance overall, the few items within each instrument that were found to be relevant to assessing the quality of health research reports were also included in this pool of items. Using items from previously developed tools is advantageous in that these items have already undergone some degree of validation and have demonstrated acceptable psychometric performance [13–15]. Items were phrased as descriptive statements, which raters can endorse to varying degrees, depending on the extent to which the statements apply to the media report being evaluated.

To ensure content coverage, throughout the item generation process, items were mapped onto a matrix with each row representing an item and each column representing one of five themes that emerged from interviews. At least five items were generated for each of the five themes (range: 5-13).

A final global item, which queries the overall quality of the media report and is to be interpreted independently of the overall score, was also developed. Such a global item is useful in cases where the quality of a media report is more nuanced than can be captured by defined criteria [16]. This is occasionally seen with assessments of competence where global rating scales can demonstrate superior reliability, compared to lengthier scales.

### Item reduction

It was decided that the instrument should include approximately 15 to 20 items to reduce burden on respondents, who for research or monitoring purposes, may be required to evaluate a large number of media reports, while also ensuring that the scale would be able to adequately discriminate between media reports of varying quality and would demonstrate acceptable reliability. Additionally, the number of items within similar instruments like the EQIP and DISCERN fall within this range.

Items generated by the investigators were reviewed with a group of five key informants (three health researchers and two health journalists who did not participate in the item generation process) who were instructed to review items for clarity and relevance. Items found to be unclear or irrelevant were excluded. To ensure that all themes were adequately covered, items were again mapped onto a matrix. Each domain included at least three items.

### Formatting and response options

To facilitate ease of administration, items and domains were ordered according to the likely order of presentation of corresponding elements in media reports required for their assessment. A seven-point adjectival scale was used for all items. For each response option, an adjective which matched the item stem was selected and all response options were numbered. A seven-point scale was selected to maximize reliability and sensitivity and to compensate for potential end-aversion bias, while also avoiding cognitive overload in prospective respondents [17–19]. A seven-point scale has also performed well for other quality assessment instruments. For example, the Appraisal of Guidelines for Research & Evaluation II (AGREE II) instrument for assessing the quality of clinical practice guidelines also utilizes a seven-point scale and has demonstrated acceptable reliability [20].

### Scoring

The QIMR is scored by adding the numbers corresponding to each endorsed response option together. Scores can be reported for each domain and/or totaled across all domains. We encourage prospective users to report scores as a percentage of the maximum possible attainable score in a domain or the full scale to aid interpretability.

### Sample

The objective of the sampling strategy was to obtain a sample of media reports of variable quality, reflective of the content to which the public is regularly exposed. The selection of news sources which would yield artificially high variation in quality and inflated reliability coefficients was avoided. A purposive sample of four Canadian news sources (The Toronto Star, National Post, The Hamilton Spectator, and Winnipeg Sun) of varying rates of circulation was selected. Factiva (https://global.factiva.com) was used to search the four news sources for health research reports according to the search strategy presented in Additional file 2, developed with assistance from a social science research librarian. The search was conducted on March 9, 2016. Two investigators (DZ and MO) selected the seven most recent media reports published by each news source that met the predefined inclusion criteria. Media reports that focused predominantly on a health research study were included (i.e., half or more of the word count was dedicated to the reporting of a health-related research study). Letters to the editor, media reports on research findings not published in peer-reviewed journals, and media reports which discussed findings from more than two research studies were excluded. Conflicts between investigators regarding the inclusion status of a media report were resolved by discussion.

### Rating the quality of media reports

Two investigators (DZ and MO) independently evaluated the quality of the included media reports using the QIMR and the accompanying manual.

### Statistical analysis

Statistical analyses were performed using SPSS Version 21 (Boston, Massachusetts, 2012) and G-String Version IV (http://fhs.mcmaster.ca/perd/index.html).

#### Item and domain characteristics

Item and domain characteristics, including measures of central tendency (i.e., means, medians), dispersion (i.e., standard deviations), and internal consistency (i.e., Cronbach’s alpha coefficients) were computed. Corrected item-total correlations were also calculated by correlating item scores with the total QIMR score to evaluate the scale for unidimensionality.

#### Scale reliability

To test the reliability of the QIMR, Generalizability theory (G theory) was used [21]. In contrast to classical test theory, G theory has the advantage of allowing assessment of the relative contribution of variance across multiple sources, referred to as facets of generalization, simultaneously, thus producing more accurate reliability estimates. G studies yield relative (i.e., norm-referenced) and absolute (i.e., criterion referenced) G coefficients, which can be interpreted similar to reliability coefficients. To compute G coefficients, facets of generalization are designated as either fixed or random. Random facets contribute to the error term in the calculation of the reliability coefficient and fixed facets do not. In this investigation, the object of differentiation was specified as media reports. Three facets of generalization were identified: item nested within domain, domain, and rater.

Five generalizability coefficients were calculated: an internal consistency generalizability coefficient was computed by holding rater and domain fixed and designating item as a random facet of generalization; an inter-domain generalizability coefficient where raters and items were fixed and domains were designated as a random facet of generalization; three inter-rater generalizability coefficients by varying the number of raters and holding items and domains fixed and designating raters as a random facet of generalization; and an overall mixed generalizability coefficient was calculated by designating all facets as random. Relative inter-item and inter-domain generalizability coefficients were interpreted under the assumption that items and domains will not vary between administrations of the QIMR, whereas absolute inter-rater generalizability coefficients were interpreted under the assumption that the raters used to generate the data are a random sample of all possible raters and systematic differences between raters is important in interpreting QIMR scores. The overall generalizability coefficient was calculated as a mixed coefficient to retain the systematic effects of raters and remove the systematic effects of items and domains. Traditionally, for non-clinical or high-stake measurement, reliability coefficients below 0.5 are seen as unreliable, measures between 0.5 and 0.7 are modest, and reliability coefficients above 0.7 indicate satisfactory reliability [22]. This standard was used to interpret reliability and generalizability coefficients with the caveat that these criteria are less stringent for generalizability coefficients, which take into account multiple sources of variation simultaneously.

#### Construct validation

Pearson correlation coefficients were used to test two convergent construct validity hypotheses: the correlation of the QIMR total score with raters’ global impression and media report word count. Although the QIMR is completed on an adjectival scale, scores generated from Likert or adjectival scales generally demonstrate interval properties and parametric tests are robust against these interval assumptions [23]. Hence, Pearson correlation coefficients were thought to be appropriate.

#### Sample size

Given that reliability coefficients are not substantially impacted by sample size and the absence of existing guidelines for conducting sample size calculations for G studies [16], a sample size of 28 media reports was pragmatically chosen. Assuming a strong correlation of 0.8 between global and QIMR score, we estimated that 28 media reports would provide more than sufficient power at the 0.05 alpha level. Assuming a moderate correlation of 0.5 between word count and QIMR score, a sample size of 28 media reports would provide 80% power to detect a statistically significant correlation at the 0.05 alpha level.

## Results

### Media report characteristics

Characteristics of the 28 included media reports and the four included news sources are presented in Tables 1 and 2. Media reports described studies from a range of medical specialties. The three most commonly reported specialties were public health (5; 18%), neonatology and pediatrics (5; 18%), and infectious disease (4; 14%). The mean word count of included media reports was 587.21 (SD 290.49) and ranged from 127 to 1175 words. The mean QIMR rating was 50.86% (SD 15.64%).

**Table 1:** Media report characteristics

Specialty^a^	Number of articles (%)
Cardiology/Vascular disease	2 (7.14)
Diet	1 (3.57)
Emergency medicine	2 (7.14)
Endocrinology	2 (7.14)
Gastroenterology	1 (3.57)
Gynecology	1 (3.57)
Infectious disease	4 (14.29)
Neonatology/Pediatrics	5 (17.86)
Oncology	2 (7.14)
Psychiatry	2 (7.14)
Public health	5 (17.86)
Respirology	1 (3.57)
Surgery	1 (3.57)
Media report descriptives	
Mean word count (SD) [range]	587.21 (290.49) [127-1175]
Mean QIMR rating, excluding global rating (/102) (SD) {%} [range]	51.88 (15.95) {50.86%} [20-84]
Mean global score (SD) (/7) [range]	4.21 (1.47) [2–6]

^a^Media reports may fit under multiple specialties

**Table 2:** News source characteristics

	Toronto Star	National Post	The Spectator	Winnipeg Sun
Number of articles	7	7	7	7
Median QIMR rating (excluding global rating) (/102) (%) [range]	62 (60.78%) [20-84]	49.5 (48.53%) [37-68]	54.5 (53.43%) [24-75]	50.5 (49.51%) [24-72]
Median ‘background’ domain score (/35) (%) [range]	25.5 (72.86%) [11-30]	18.5 (52.86%) [11-25]	16 (45.71%) [10-28]	16.5 (47.14%) [8-25]
Median ‘sources’ domain score (/21) (%) [range]	12.5 (59.52%) [0-18]	7 (33.33%) [1–15]	8 (38.10%) [1–14]	9 (42.86%) [3–16]
Median ‘results’ domain score (/21) (%) [range]	15 (71.43%) [5–18]	13 (61.90%) [6–18]	12 (57.14%) [4–18]	11 (52.38%) [4–18]
Median ‘context’ domain score (/21) (%) [range]	11.5 (54.76%) [0-18]	9.5 (45.24%) [3–18]	11 (52.38%) [3–18]	8 (38.10%) [3–15]
Median ‘validity’ domain score (/21) (%) [range]	0 (0%) [0-14]	0 (0%) [0-16]	4 (19.05%) [0-11]	0 (0%) [0-4]
Median global score (/7) [range]	5 [2-6]	4 [2-6]	4 [2-6]	3 [2-6]

### Item characteristics

Item characteristics are presented in Table 3. Responses for most items appear to be approximately normally distributed, as indicated by similar mean and median statistics. All item standard deviations exceed one, indicating variability in responses. Response options endorsed appear to range the full scale, as illustrated by the minimum and maximum values. Endorsement frequencies of response options for all items were individually examined. No response option for any of the items produced an endorsement frequency greater than 75%. These results suggest that all items are adequately differentiating between media reports.

**Table 3:** Item statistics

Item	Mean	SD	Median	Mode	Minimum	Maximum	Item-total correlation
1a	3.45	1.82	3	2	0	6	0.38
1b	3.16	2.02	3	3	0	6	0.64
1c	4.39	1.77	5	6	0	6	0.15
1d	4.18	1.73	5	6	1	6	*0.11*
1e	3.21	1.93	3	6	0	6	0.56
2a	3.57	1.80	4	4	0	6	*0.23*
2b	1.75	2.40	0	0	0	6	0.36
2c	3.57	1.77	3	3	0	6	0.62
3a	4.23	1.67	5	6	1	6	0.56
3b	3.57	1.93	4	6	0	6	0.58
3c	4.16	1.75	4	6	1	6	0.48
4a	3.77	1.65	4	6	0	6	0.62
4b	2.70	2.50	3	0	0	6	0.37
4c	3.45	1.88	4	4	0	6	0.59
5a	0.93	1.73	0	0	0	6	*0.14*
5b	1.04	1.76	0	0	0	6	0.35
5c	0.75	1.52	0	0	0	6	*0.23*
GLOBAL	4.21	1.47	4	3	2	6	

The italicized figures indicate item-total correlation coefficients outside the accepted range

Most items appear to have acceptable item-total correlation values, between 0.3 and 0.7 [16]. Four items were poorly correlated with total scale scores: one item in the ‘background’ domain assessing use of jargon, one item in the ‘sources’ domain assessing the identification of the organizational and financial affiliations of the study, and two items in the ‘validity’ domain assessing whether the appropriateness of the study methodology and strengths and weaknesses of the study are adequately discussed.

### Domain characteristics

Domain statistics are presented in Table 4. The mean domain scores ranged from 15.06% to 66.41% for the ‘validity’ and ‘results’ domains, respectively. Standard deviation statistics ranged from 2.26 points (12.56%) to 4.44 points (24.67%) for the ‘results’ and ‘context’ domains, respectively.

**Table 4:** Domain Statistics

Domain	Number of items	Mean (%)	SD (%)	Range	Cronbach’s Alpha
Background (/35)	5	18.39 (52.54)	5.67 (18.90)	8-30	*0.58*
Sources (/18)	3	8.89 (49.39)	4.25 (23.61)	0-18	*0.49*
Results (/18)	3	11.96 (66.44)	2.26 (12.56)	4-18	0.71
Context (/18)	3	9.91 (55.06)	4.44 (24.67)	0-18	*0.55*
Validity (/18)	3	2.71 (15.06)	4.43 (24.61)	0-16	0.86

The italicized figures indicate item-total correlation coefficients outside the accepted range

The ‘background’, ‘sources’, and ‘context’ domains produced less than ideal Cronbach’s alpha coefficients, ranging from 0.49 for the ‘sources’ domain to 0.58 for the ‘background’ domain. Cronbach’s alpha values for the remaining two domains were above 0.70.

### Generalizability study

Variance components from the G study are presented in Table 5. The three largest sources of variance were media reports (38.85%), domains (29.21%), and the interaction between media reports and raters (11.26%).

**Table 5:** Variance components generated from the Generalizability study

Source	Variance Component	Levels	%
Media reports	0.344	1	38.35
Rater	0	3	0.00
Domain	0.262	4.74	29.21
Item:Domain	0.019	16.12	2.12
Media reports * Rater	0.101	3	11.26
Media reports * Domain	0.084	4.74	9.36
Media reports * Item:Domain	0.039	16.12	4.35
Rater * Domain	0.003	3.00*4.74	0.33
Rater * Item:Domain	0	3.00*16.12	0.00
Media reports * Rater * Domain	0.009	3.00*4.74	1.00
Media reports * Rater * Item:Domain	0.036	3.00*16.12	4.01

The * is a notation used in g-theory to indicate interaction

Generalizability coefficients are presented in Table 6. The internal consistency of the scale was estimated at 0.87, inter-domain reliability was 0.72, and inter-rater reliability between two raters was 0.68. Averaging QIMR ratings over three raters increased the inter-rater reliability to 0.76. The overall generalizability coefficient for the scale was 0.54.

**Table 6:** Generalizability coefficients generated from G study

Rater	Domain	Item	Absolute Coefficient	Relative Coefficient	Interpretation
Fixed	Fixed	Random	0.84	**0.87**	Internal consistency
Fixed	Random	Fixed	0.51	**0.72**	Inter-domain reliability
Random (2 raters)	Fixed	Fixed	**0.68**	0.68	Inter-rater reliability
Random (3 raters)	Fixed	Fixed	**0.76**	0.76	Inter-rater reliability
Random (4 raters)	Fixed	Fixed	**0.81**	0.81	Inter-rater reliability
Random (3 raters)	Random (main effects removed)	Random (main effects removed)	**0.54**		overall, adjusted for multiple (3) raters

Bolded values indicate whether absolute or relative coefficients should be interpreted

### Construct validity

**Fig. 2:**
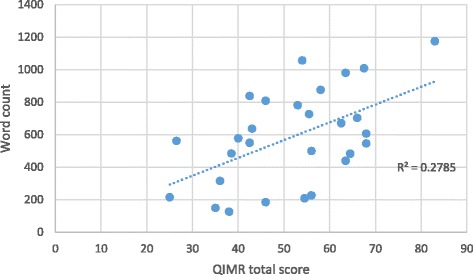
Relationship between total QIMR score and media report word count

**Fig. 3:**
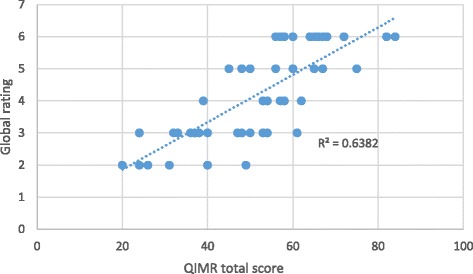
Relationship between QIMR total scores and global rating

Table 7 presents the results of the constructs tested. QIMR scores were highly correlated with raters’ global impression and moderately correlated with word count. The relationship between QIMR scores and global ratings and word count are presented in Figs. 2 and 3, respectively.

**Table 7:** Construct validation results (Pearson correlation coefficients)

Correlation with word count	Correlation with global rating
0.528 (*p* = 0.004)	0.799 (*p* < 0.001)

## Discussion

The results from the preliminary reliability and validity testing suggest that the QIMR demonstrates adequate reliability and validity for use by health researchers to evaluate the quality of health research reports in the lay media.

### Media report characteristics

A diverse range of media reports were included in this analysis. Included media reports varied in length, were published in news sources with varying rates of circulation, and reported on research articles from a range of medical specialties. Diversity in the object of measurement is a necessary precondition for testing reliability and correlating scores with other measures to establish construct validity [16]. Finally, establishing reliability and validity in a diverse sample allows potential users to be confident in applying the QIMR to a wider range of media reports.

### Item characteristics

Most QIMR items performed satisfactorily. All items generally produced normal response distributions. Floor effects were detected for items in the ‘validity’ domain of the QIMR. Modifying response options was considered to deal with this effect. Closer examination of these items revealed that all response options were used for at least one media report and the probability of endorsing any single response option did not exceed 75%. This suggests adequate variability in responses, which we concluded did not warrant modification to the scale.

Most items on the QIMR demonstrated acceptable correlation with the total scale score. This suggests that the scale is measuring a singular construct with low item redundancy. Four items (1d, 2a, 5a, 5c) produced item-total correlations less than 0.3, suggesting that they may be measuring characteristics of media reports other than quality. Closer examination of these items revealed good face validity. It was hypothesized that these items measured characteristics that were conceptually relevant to the overall quality of media reports but not necessarily highly correlated with other indicators of quality. The poor performance of these items may also be an artifact of the sample of media reports evaluated in this investigation. Testing an additional sample of media reports and evaluating raters’ interpretation of these items may shed light on these results.

### Domain characteristics

The domains of the QIMR demonstrated good score variability, suggesting that domains have acceptable ability to discriminate between media reports of varying quality. The last domain of the QIMR, the ‘validity’ domain, produced a negatively skewed score distribution with a floor effect. The domain was retained as it produced adequate variability in scores. Furthermore, as items and domains included in the QIMR emerged from interviews with key informants, it was decided that eliminating the ‘validity’ domain may reduce the content validity of the instrument. We encourage users of the QIMR to exercise caution when using and interpreting ‘validity’ domain scores, as this domain may lack adequate sensitivity.

Three domains produced lower than ideal Cronbach’s alpha values, suggesting that they may not be unidimensional. However, this is most likely due to the inclusion of few items within each domain.

### Generalizability study

The G study suggests that QIMR scores have moderate generalizability for use as a research tool. As expected, media reports accounted for the most observed variance, indicating that the QIMR is sensitive to the quality of individual media reports. Domains also accounted for significant variance, most likely due to each domain is evaluating an independent, singular characteristic of media reports. The variability observed due to the interaction between media reports and raters is larger than ideal. Averaging QIMR ratings over a larger number of raters should improve generalizability.

The scale demonstrated good internal consistency. Inter-domain generalizability, which can be interpreted as the extent to which ratings on one domain can be generalized to another domain, was moderate. There was also moderate inter-rater generalizability, which can be interpreted as the extent to which ratings by one rater can be generalized to another rater. Increasing the number of raters to three yielded acceptable inter-rater generalizability. We recommend for QIMR scores to be averaged over at least three raters to obtain stable and reliable quality scores.

### Construct validity

The construct validation testing confirmed the hypothesized relationships between total QIMR scores and raters’ global assessment of media reports and word count. A strong (0.80) relationship was detected between QIMR scores and raters’ global assessment of media reports and a moderate (0.53) relationship between QIMR scores and media report length was detected. These results lend credence to the validity of the QIMR for measuring the quality of health research reports in the media and the theory linking raters’ global assessment and word count to the overall quality of media reports.

### Limitations and future directions

This investigation is not without limitations. The potential non-representativeness of the sample of health researchers used to develop the theoretical framework according to which items were generated must be acknowledged. The sample of health journalists who agreed to participate in this investigation may have also been systematically different from other journalists. These journalists may have been highly motivated or interested in publishing high quality content. Similarly, the health researchers interviewed during the item generation process were all members of one institution and so may have shared a similar perspective on the topic.

In evaluating the reliability of the QIMR, it must also be noted that some level of subjectivity is always involved in judging the adequacy of reliability coefficients. Furthermore, interpretation of generalizability coefficients is less straightforward than the interpretation of standard reliability coefficients. Because generalizability theory allows modeling of multiple sources of error which impact reliability, it is likely that values of generalizability coefficients might only approach the levels considered acceptable in classical test theory. Thus, the overall generalizability coefficient obtained for the QIMR, while slightly lower than reliability coefficients considered adequate in classical test theory, may in fact indicate acceptable reliability.

Finally, the evaluation of the quality of health research reports in the media is influenced by the content knowledge of the raters and their familiarity with the topic being reported. It may be of interest to evaluate the performance of the QIMR for use by raters with more or less health research experience.

Areas for future research may include testing the sensitivity of the QIMR to news sources. It is likely that news sources will publish content of varying quality. Differences were observed in QIMR total and domain scores for the four different news sources. However, no statistical tests were conducted to evaluate this relationship due to our limited sample size. Future research may also be directed at assessing the performance of the QIMR for evaluating health research reports published in American or European news sources.

## Conclusions

The QIMR demonstrated adequate validity and reliability for use as a tool to evaluate the quality of health research reporting in the media. This tool can be used for research purposes to identify correlates of high and poor quality reporting of medical research. It can also be used to identify areas of weakness in health research reports and to subsequently develop targeted interventions to improve the state of health research reporting. It is anticipated that monitoring and improving the state of health research reporting may lead to the better utilization of the media as a public health tool.

## Additional files

Additional file 1: Quality Index for health-related Media Reports (QIMR). (DOC 87 kb)

Additional file 2: Search strategy for reports on health-related research in the media ran on Factiva. (DOCX 41 kb)

## Abbreviations

G study: Generalizability study
G theory: Generalizability theory
QIMR: Quality Index for health-related Media Reports
