# Supplementary material for: The development and validation of an instrument to measure the quality of health research reports in the lay media
Source: BMC Public Health. 2017 Apr 20;17:343. doi: 10.1186/s12889-017-4259-y (PMC5397754; doi:10.1186/s12889-017-4259-y)
Supplement: Supplementary file 1 — Quality Index for health-related Media Reports (QIMR). (DOC 87 kb) [file 12889_2017_4259_MOESM1_ESM.doc]

| **Quality Index for health-related Media Reports (QIMR)** | | | | | | | | | | |
| --- | --- | --- | --- | --- | --- | --- | --- | --- | --- | --- |
| **General Information** | | | | | | | | | | |
| Title | | | |  | | | | | | |
| Author | | | |  | | | | | | |
| News source | | | |  | | | | | | |
| Date of publication | | | |  | | | | | | |
| **Quality Assessment** Please indicate the degree to which the below statements apply to the media report using the provided scales. | | | | | | | | | | |
| 1. Background | | | | | | | | | | |
| a. Rationale for the study is clearly explained. | | | | | | | | | | |
| Not at all | To a very limited extent | To a limited extent | To a moderate extent | | | To a great extent | To a very great extent | | Completely | |
| 1 | 2 | 3 | 4 | | | 5 | 6 | | 7 | |
| b. The state of the field (e.g., what is currently known about the topic) is described. | | | | | | | | | | |
| Not at all | To a very limited extent | To a limited extent | To a moderate extent | | To a great extent | | | To a very great extent | | Completely |
| 1 | 2 | 3 | 4 | | 5 | | | 6 | | 7 |
| c. Technical concepts are accessible to the lay public. | | | | | | | | | | |
| Not at all | To a very limited extent | To a limited extent | To a moderate extent | | | To a great extent | To a very great extent | | Completely | |
| 1 | 2 | 3 | 4 | | | 5 | 6 | | 7 | |
| d. Use of jargon is avoided or jargon is defined for lay readers. | | | | | | | | | | |
| Not at all | To a very limited extent | To a limited extent | To a moderate extent | | | To a great extent | To a very great extent | | Completely | |
| 1 | 2 | 3 | 4 | | | 5 | 6 | | 7 | |
| e. The headline accurately reflects research findings. | | | | | | | | | | |
| Not at all | To a very limited extent | To a limited extent | To a moderate extent | | | To a great extent | To a very great extent | | Completely | |
| 1 | 2 | 3 | 4 | | | 5 | 6 | | 7 | |
| 2. Sources | | | | | | | | | | |
| a. Institution(s)/organization(s) affiliated with the study is/are identified. | | | | | | | | | | |
| Not at all | To a very limited extent | To a limited extent | To a moderate extent | | | To a great extent | To a very great extent | | Completely | |
| 1 | 2 | 3 | 4 | | | 5 | 6 | | 7 | |
| b. Relevant, informative, and qualified expert(s), excluding original study investigators, were consulted. | | | | | | | | | | |
| Not at all | To a very limited extent | To a limited extent | To a moderate extent | | | To a great extent | To a very great extent | | Completely | |
| 1 | 2 | 3 | 4 | | | 5 | 6 | | 7 | |
| c. Opinions and facts are clearly distinguished. | | | | | | | | | | |
| Not at all | To a very limited extent | To a limited extent | To a moderate extent | | | To a great extent | To a very great extent | | Completely | |
| 1 | 2 | 3 | 4 | | | 5 | 6 | | 7 | |
| 3. Results | | | | | | | | | | |
| a. Conclusion(s)/Results of the study are clearly and accurately communicated. | | | | | | | | | | |
| Not at all | To a very limited extent | To a limited extent | To a moderate extent | | | To a great extent | To a very great extent | | Completely | |
| 1 | 2 | 3 | 4 | | | 5 | 6 | | 7 | |
| b. All important research findings are reported, regardless of possible contradictions between different outcomes. | | | | | | | | | | |
| Not at all | To a very limited extent | To a limited extent | To a moderate extent | | | To a great extent | To a very great extent | | Completely | |
| 0 | 1 | 2 | 3 | | | 4 | 5 | | 6 | |
| c. All claims are supported by research findings. | | | | | | | | | | |
| Not at all | To a very limited extent | To a limited extent | To a moderate extent | | | To a great extent | To a very great extent | | Completely | |
| 0 | 1 | 2 | 3 | | | 4 | 5 | | 6 | |
| 4. Context | | | | | | | | | | |
| a. An accurate interpretation of the results of the study is provided. | | | | | | | | | | |
| Not at all | To a very limited extent | To a limited extent | To a moderate extent | | | To a great extent | To a very great extent | | Completely | |
| 0 | 1 | 2 | 3 | | | 4 | 5 | | 6 | |
| b. Consistency of results with previous findings are discussed. | | | | | | | | | | |
| Not at all | To a very limited extent | To a limited extent | To a moderate extent | | | To a great extent | To a very great extent | | Completely | |
| 0 | 1 | 2 | 3 | | | 4 | 5 | | 6 | |
| c. Clinical and/or real-world significance of the results have been accurately identified. | | | | | | | | | | |
| Not at all | To a very limited extent | To a limited extent | To a moderate extent | | | To a great extent | To a very great extent | | Completely | |
| 0 | 1 | 2 | 3 | | | 4 | 5 | | 6 | |
| 5. Validity | | | | | | | | | | |
| a. Appropriateness of the study methodology in answering the research question is discussed. | | | | | | | | | | |
| Not at all | To a very limited extent | To a limited extent | To a moderate extent | | | To a great extent | To a very great extent | | Completely | |
| 0 | 1 | 2 | 3 | | | 4 | 5 | | 6 | |
| b. Well-founded evaluation of the validity of the evidence is provided. | | | | | | | | | | |
| Not at all | To a very limited extent | To a limited extent | To a moderate extent | | | To a great extent | To a very great extent | | Completely | |
| 0 | 1 | 2 | 3 | | | 4 | 5 | | 6 | |
| c. A balanced description of the strengths and weaknesses of the study is provided. | | | | | | | | | | |
| Not at all | To a very limited extent | To a limited extent | To a moderate extent | | | To a great extent | To a very great extent | | Completely | |
| 0 | 1 | 2 | 3 | | | 4 | 5 | | 6 | |
| **Global Rating** | | | | | | | | | | |
| Taking into consideration the above assessment of the media report and any of its additional relevant attributes, please rate the overall quality of the report. | | | | | | | | | | |
| The message communicated in the media report is explicitly incorrect | Three or four problems that undermine the main message of the report | One or two problems that undermine the main message of the report | One or two problems that do not undermine the main message of the report | | | Informative with one or two minor problems that do not undermine the message of the report | Very informative with one or two minor problems | | Very informative with no problems | |
| 0 | 1 | 2 | 3 | | | 4 | 5 | | 6 | |
