# Supplementary material for: The development and validation of an instrument to measure the quality of health research reports in the lay media
Source: BMC Public Health. 2017 Apr 20;17:343. doi: 10.1186/s12889-017-4259-y (PMC5397754; doi:10.1186/s12889-017-4259-y)
Supplement: Supplementary file 2 — Search strategy for reports on health-related research in the media ran on Factiva. (DOCX 41 kb) [file 12889_2017_4259_MOESM2_ESM.docx]

| Search strategy for reports on health-related research in the media ran on Factiva | | |
| --- | --- | --- |
| (new research or new study or study published or research published or journal or annals or archives) AND (health* or medic* or treatment* or surg* or drug* or diagnos* or screening) | | |
| News sources |  | Toronto Star, National Post, The Spectator, Winnipeg Sun |
| Date | | In the last 5 years |
| Subject | | Health |
